# Performance of T-Track^®^ SARS-CoV-2, an Innovative Dual Marker RT-qPCR-Based Whole-Blood Assay for the Detection of SARS-CoV-2-Reactive T Cells

**DOI:** 10.3390/diagnostics13172722

**Published:** 2023-08-22

**Authors:** Franziska M. Kanis, Johannes P. Meier, Harald Guldan, Hans-Helmut Niller, Michael Dahm, Alexander Dansard, Thomas Zander, Friedhelm Struck, Erwin Soutschek, Ludwig Deml, Selina Möbus, Sascha Barabas

**Affiliations:** 1 Mikrogen GmbH, 82061 Neuried, Germany; 2 Institute for Medical Microbiology and Hygiene, University of Regensburg, 93053 Regensburg, Germany; 3 Aureleon, 81677 Munich, Germany; 4 nal von minden GmbH, 93053 Regensburg, Germany

**Keywords:** T-Track^®^ SARS-CoV-2, COVID-19, T cells, T-cell immunity, RT-qPCR, *CXCL10*, *IFNG*, SARS-CoV-2, S1 antigen, NP antigen

## Abstract

T-cell immunity against severe acute respiratory syndrome coronavirus 2 (SARS-CoV-2) plays a central role in the control of the virus. In this study, we evaluated the performance of T-Track^®^ SARS-CoV-2, a novel CE-marked quantitative reverse transcription-polymerase chain reaction (RT-qPCR) assay, which relies on the combined evaluation of *IFNG* and *CXCL10* mRNA levels in response to the S1 and NP SARS-CoV-2 antigens, in 335 participants with or without a history of SARS-CoV-2 infection and vaccination, respectively. Of the 62 convalescent donors, 100% responded to S1 and 88.7% to NP antigens. In comparison, of the 68 naïve donors, 4.4% were reactive to S1 and 19.1% to NP. Convalescent donors <50 and ≥50 years of age demonstrated a 100% S1 reactivity and an 89.1% and 87.5% NP reactivity, respectively. T-cell responses by T-Track^®^ SARS-CoV-2 and IgG serology by recomLine SARS-CoV-2 IgG according to the time from the last immunisation (by vaccination or viral infection) were comparable. Both assays showed a persistent cellular and humoral response for at least 36 weeks post immunisation in vaccinated and convalescent donors. Our results demonstrate the very good performance of the T-Track^®^ SARS-CoV-2 molecular assay and suggest that it might be suitable to monitor the SARS-CoV-2-specific T-cell response in COVID-19 vaccinations trials and cross-reactivity studies.

## 1. Introduction

Several studies indicate that, besides antibody-mediated response, T cell response is a significant component of immune protection against severe acute respiratory syndrome coronavirus 2 (SARS-CoV-2) [1,2,3,4]. SARS-CoV-2-specific T cells are usually detectable a few days after viral infection [5,6], and their evaluation might serve as a predictor of future disease severity [7,8]. Since SARS-CoV-2-specific antibody titres often drop several months after infection [9,10,11], and SARS-CoV-2-specific T cells have been detected in the absence of virus-specific antibodies in exposed persons [12,13,14], there is a considerable public interest and clinical relevance in evaluating T cell-mediated immunity in individuals exposed to SARS-CoV-2, as well as following vaccination against SARS-CoV-2.

T-cell responses are detectable in almost all SARS-CoV-2 infected and vaccinated individuals [12,15,16,17]. T cell responses comprise T helper (Th) cells predominantly of the Th1-type and cytotoxic T cells (CTL) with a wide range of effector functions and are directed against a broad spectrum of target structures, particularly within the spike 1 (S1), nucleo (N) and matrix (M) protein [15,18,19]. While the SARS-CoV-2-specific antibody response is particularly important in preventing infection and reducing the infectious dose, the T-cell response supports replication control and elimination of the virus and appears to attenuate the pathology associated with COVID-19 disease. Recent studies show that individuals who mount a strong SARS-CoV-2-specific T cell-response in the early post-infection phase usually have a mild disease course and accelerated viral clearance [20,21]. Another indication of the protective properties of cell-mediated immunity is that patients with agammaglobulinaemia are able to control SARS-CoV-2 infections without severe disease symptoms [22]. Compared to humoral immunity, the T cell response also appears to be more stable over time after vaccination or recovery and exhibits higher cross-reactivity to newly emerging viral variants [23]. Furthermore, in individuals vaccinated with RNA vaccines, early protection against COVID-19 is mediated by early T-cell responses rather than neutralizing antibodies [24].

Numerous tests have been developed to measure SARS-CoV-2-specific T-cell responses. Stimulation of T cells using SARS-CoV-2 antigens and measurement of interferon-gamma (IFN-γ) released by antigen-specific T cells through already commercially available interferon-gamma release assays (IGRAs) such as T-SPOT^®^.COVID (Oxford Immunotec) [25], Quantiferon^®^ SARS-CoV-2 (Qiagen, Hilden, Germany) [26], Quan-T-Cell (Euroimmun, Lübeck, Germany) [27], DuoColour T-Cellspot^®^ SARS-CoV-2 (Euroimmun, Lübeck, Germany) and CoV-/Spot SARS-CoV-2 FluoroSpot (AID, Straßberg, Germany), have become the most widespread option for the evaluation of SARS-CoV-2-specific T cell immunity. As an advancement, the last two mentioned IGRAs measure a second biomarker, interleukin 2 (IL-2), which is characteristic of memory T cells. Compared to all mentioned IGRAs, the unique feature of the novel CE-marked T-Track^®^ SARS-CoV-2 assay (Mikrogen, Neuried, Germany) is the application of quantitative reverse transcription-polymerase chain reaction (RT-qPCR) and the combined evaluation of the mRNA biomarkers *IFN-γ* and *CXCL10* as a measure of T cell reactivity to two relevant SARS-CoV-2 protein antigens, the S1 subunit of the spike protein (S1) and the nucleocapsid protein (NP), separately.

The objective of this study was to analyse T-cell responses using T-Track^®^ SARS-CoV-2 in participants with or without a history of SARS-CoV-2 exposure (by infection or vaccination).

## 2. Materials and Methods

### 2.1. Study Design and Participants

Blood samples were collected from volunteers with and without a history of SARS-CoV-2 infection at the Institute for Medical Microbiology and Hygiene, University of Regensburg, Germany, and the Allgemeinmedizinische Praxis im Posthof, Regensburg, Germany. Subjects below 18 years of age, pregnant women, as well as individuals with an inability or unwillingness to provide informed consent, known to be infected with human immunodeficiency virus (HIV), hepatitis B virus (HBV), or hepatitis C virus (HCV), and patients under immunosuppressive therapy, were excluded from enrolment. Samples were categorised into five groups according to the donors’ SARS-CoV-2 immunisation status. The first group included donors naïve for SARS-CoV-2, defined as non-vaccinated donors with no history of a SARS-CoV-2 infection and with negative recomLine SARS-CoV-2 IgG S1 and NP results. Vaccinated individuals with a negative recomLine SARS-CoV-2 IgG NP test result were categorized into three groups, with one, two, or three immunisations, respectively, using a spike mRNA-based vaccine (BNT162b2 (Comirnaty^®^, BioNTech, Germany/Pfizer, New York, NY, USA), mRNA-1273 (Spikevax^®^, Moderna Biotech, MA, USA), or a combination thereof). This ensured that donors had been exposed to a comparable source of antigen (notably the S1 antigen); a negative recomLine SARS-CoV-2 IgG NP results further ensured that these vaccinated donors had no history of a SARS-CoV-2 infection. Vaccinated donors with blood withdrawn 14–275 days after vaccination were included in the analysis. The shorter time point of 14 days was chosen because high numbers of IFNG-secreting cells were detected within 15 days of symptom onset in most SARS-CoV-2-infected patients [28]. The maximum time point of 275 days was selected to ensure that a T cell response was detectable, based on reports demonstrating the maintenance of SARS-CoV-2-specific cellular and humoral immunity one year after disease onset [29,30,31]. The fifth group included COVID-19 convalescent participants (thus with a history of SARS-CoV-2 infection and exposure to multiple SARS-CoV-2 antigens, including S1 and NP), with blood withdrawn 14–275 days after SARS-CoV-2 infection diagnosis (confirmed by molecular testing), regardless of their vaccination and recomLine SARS-CoV-2 IgG serology status.

The study was approved by the Ethics Committee of the Bayerischen Landesärztekammer (BLÄK), Munich, Germany (reference number 22040). Written consent was obtained from all individuals enrolled in the study.

### 2.2. Sample Collection

Blood samples were collected in two types of monovettes: in lithium-heparin collection tubes (Sarstedt S-Monovette^®^ 7.5 mL LH, Nümbrecht, Germany; cat. No. 01.1604.001) for T-Track^®^ SARS-CoV-2 testing, and in clotting activator-containing monovettes (Sarstedt S-Monovette^®^ 7.5 mL Z; cat. No. 01.1601.001) for recomLine SARS-CoV-2 IgG testing. Samples collected at different locations (Institute for Medical Microbiology and Hygiene, University of Regensburg, Germany, and the Allgemeinmedizinische Praxis im Posthof, Regensburg, Germany) were transported by courier at room temperature (18–25 °C) within 8 h of withdrawal to the two measuring laboratories at Mikrogen GmbH (Regensburg and Neuried, Germany). The blood samples for recomLine SARS-CoV-2 IgG were stored upright at room temperature (18–25 °C; light protected) for at least 30 min. T-Track^®^ SARS-CoV-2 and recomLine SARS-CoV-2 IgG were applied as described in the manufacturer’s instructions.

### 2.3. T-Track^®^ SARS-CoV-2 Assay

T-Track^®^ SARS-CoV-2 consists of the T-Track^®^ SARS-CoV-2 Stimulation kit (cat. No. 11001006, Mikrogen GmbH, Neuried, Germany) and the T-Track^®^ SARS-CoV-2 Quant PCR kit (cat. No. 11001007, Mikrogen GmbH). T-Track^®^ SARS-CoV-2 is a highly sensitive RT-qPCR assay for the detection of SARS-CoV-2-specific T-cell reactivity by determination of the relative expression of SARS-CoV-2 antigen-induced specific markers.

**Figure 1:**
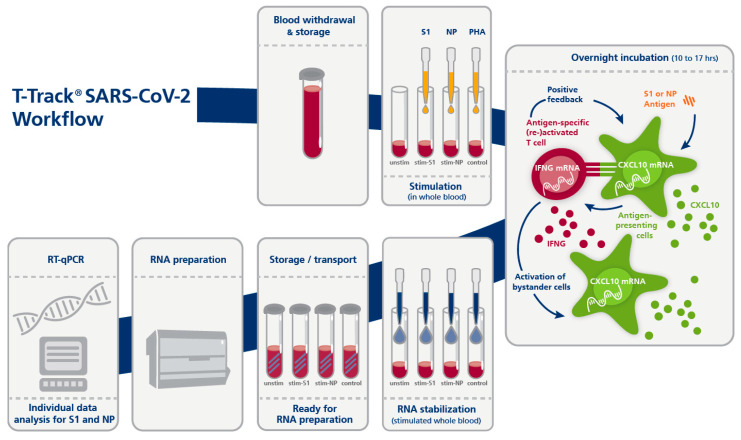
Principle and workflow of the T-Track^®^ SARS-CoV-2 assay. Abbreviations: PHA, Phytohemagglutinin; qPCR, quantitative PCR; stim-S1, stimulated with S1 antigen; stim-NP, stimulated with NP antigen; unstim, unstimulated.

The procedure of T-Track^®^ SARS-CoV-2 is very similar to the procedure of T-Track^®^ TB ([32]). T-Track^®^ SARS-CoV-2 was performed according to the manufacturer’s instructions (Figure 1). Briefly, 0.5 mL heparinized blood was transferred into four individual tubes each and stimulated with the SARS-CoV-2 antigens S1 (S1 subunit of the spike protein) or NP (nucleocapsid protein) or with PHA as stimulation control. The fourth tube served as unstimulated negative control. After addition of stimulants, the blood samples were incubated overnight (10 to 17 h) in a 37 °C incubator. RNA was then stabilized by adding T-Track^®^ RNA Stabilizer to each sample. RNA was extracted using a MagNA Pure 96 System and the MagNA Pure 96 Cellular RNA Large Volume Kit (Roche, Penzberg, Germany; cat. No. 05467535001) and stored at −80 °C until further use. RT-qPCR was performed with the T-Track^®^ SARS-CoV-2 Quant PCR kit using the extracted RNA as template. RT-qPCR was run on a QuantStudio 5 real-time PCR system (ThermoFisher Scientific, Waltham, MA, USA) using the following parameters: 10 min at 50 °C for the reverse transcription and 30 sec at 95 °C for the denaturation step, followed by 38 cycles of 5 sec at 95 °C and 30 sec at 60 °C. *IFNG*, *CXCL10,* and *RPLP0* were analysed in parallel. The increased expression of *IFNG/CXCL10* in the specifically stimulated vs. non-stimulated blood sample is an indirect marker for the presence of SARS-CoV-2-specific activated and memory T cells and indicates previous contact with SARS-CoV-2 or vaccination.

The T-Track^®^ SARS-CoV-2 assay provides a primary quantitative measuring result (the relative amount of RNA [E^(-Ct)] as calculated from the RT-qPCR Ct value) that is converted into a qualitative test result (“negative” or “positive”) by the T-Track^®^ SARS-CoV-2 Analysis Tool. This software assesses the validity of the results and classifies samples as “positive” (for detection of SARS-CoV-2 S1 and/or NP antigen-reactive T cells), “negative” (for non-detection of SARS-CoV-2 S1 and NP antigen-reactive T cells), “inconclusive” or “invalid”. The presence of S1 and NP antigen-reactive T cells is determined individually by the software.

Briefly, if the validity rules (the measured Ct values of the samples and controls must be within the defined acceptance range for all measured markers; otherwise, the sample is invalid) are met, the Ct values for *IFNG*, *CXCL10,* and *RPLP0* are corrected using their respective amplification efficiencies. The *IFNG* and *CXCL10* values (classification markers) are then normalized to the values of *RPLP0* (reference marker), and the fold change (FC) values for *IFNG* and *CXCL10* are calculated (ratio between stimulated and unstimulated states). The FC values are log2-transformed and used to generate a score (using proprietary algorithms) that allows the qualitative classification of each blood sample.

The classification threshold can be plotted as a line on a scatter plot with log2-transformed *CXCL10* FC values on the *x*-axis and log2-transformed *IFNG* FC values on the *y*-axis. Values below the threshold line are classified as negative, and values above as positive.

Distance values were calculated as the orthogonal distances of the data points (log2-transformed *CXCL10* and log2-transformed *IFNG* FC values) in this scatter plot to the threshold line. Distances of points below the threshold were given a negative sign. Classification based on the threshold and calculation of the distance is only possible if both markers are used for the analysis. In rare cases where only one marker was valid, classification was performed based on the analysis of a single marker only.

The same classification thresholds for a single marker analysis (*IFNG* and *CXCL10*) were used as those determined for the T-Track^®^ TB assay [32]. Upper and lower thresholds were defined for positivity and negativity, respectively. Single-marker FC values between the upper and lower thresholds were defined as inconclusive. Optimal upper and lower thresholds were established by minimizing the number of false negatives (FN), false positives (FP), and inconclusive results using a proprietary score formula.

### 2.4. recomLine SARS-CoV-2 IgG

The recomLine SARS-CoV-2 IgG (Mikrogen GmbH) line immunoassay is a qualitative test for the detection of SARS-CoV-2 S1, NP, and RBD antigen-specific IgG antibodies in human serum or plasma. The following recombinantly produced antigens are used in the test for SARS-CoV-2: S1, NP, and RBD (receptor binding domain of the spike protein). In addition, the test enables the detection of NP-reactive antibodies against the seasonal human coronaviruses HCoV: 229E, NL63, OC43, HKU1.

The recomLine SARS-CoV-2 IgG assay was performed according to the manufacturer’s instructions. Briefly, the nitrocellulose membrane test strips were incubated with the diluted serum sample. Unbound conjugate antibodies were subsequently washed away. Bound antibodies were detected by incubation with an anti-human IgG detection antibody coupled with horse radish peroxidase (HRP). After washing, specifically bound antibodies were detected by means of a colour reaction catalysed by the HRP.

The developed test strips were analysed using the recomScan test strip analysis software (developed by BioSciTec GmbH, Frankfurt, Germany) on the flatbed scanner Epson Perfection V39 (Epson, Los Alamitos, CA, USA). Antigen-specific band intensities were interpreted relative to the cut-off band intensity. For this study, only SARS-CoV-2 S1 and NP IgG results were used for data analysis and comparison with S1- and NP-reactive T cells detected by T-Track^®^ SARS-CoV-2.

### 2.5. Statistical Analysis

Data were analysed using Microsoft Excel for Microsoft 365 (Microsoft Corporation, Redmond, WA, USA) and GraphPad Prism 9 (Dotmatics, Boston, MA, USA).

In case of multiple samples per patient, only the first available sample (chronologically) was included in the analysis. The percentage of samples with reactive T cells in a group was defined as the ratio of the number of samples with signals above the classification threshold to the number of all samples in that group. Invalid and inconclusive test results were excluded from performance calculations. The 95% confidence interval (CI) was calculated according to Agresti and Coull [33].

The comparative analysis of the performance characteristics of T-Track^®^ SARS-CoV-2 and recomLine SARS-CoV-2 IgG was performed on samples with valid results by both methods using the McNemar test. Differences in the T-Track^®^ results according to age were analysed using Fisher’s exact test.

To compare the classification characteristics of different diagnostic markers and marker combinations, receiver operating characteristic (ROC) curves were generated. Areas under the ROC curves were compared using the method from Hanley and McNeil [34].

## 3. Results

### 3.1. Study Participants’ Characteristics

**Figure 2:**
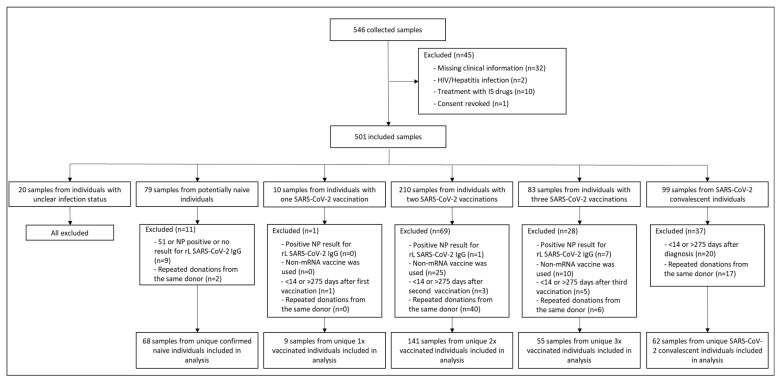
Study flow diagram. Out of 546 collected samples, 501 met the inclusion criteria, and 335 were included in the analysis, according to five clinically relevant groups: naïve for SARS-CoV-2, vaccinated once (1×), twice (2×) or three times (3×) against SARS-CoV-2, and COVID-19 convalescent donors. A total of 166 samples were excluded from the analysis due to either an unclear infection status (missing proof of SARS-CoV-2 infection in COVID-19 convalescent donors due to missing molecular diagnostic testing, contact with SARS-CoV-2-infected donors in naïve and vaccinated donors; *n* = 20), an inappropriate recomLine (rL) SARS-CoV-2 IgG result (*n* = 17), use of non-mRNA vaccine (*n* = 35), blood withdrawal out of the defined time frame (*n* = 29), or repeated donations from the same donor (*n* = 65). Abbreviations: HIV, human immunodeficiency virus; IS, immunosuppressive; MP96, MagNA Pure 96 System; pNC, processed Negative Control; rL, recomLine.

A total of 546 blood samples were collected from 332 enrolled donors. Of these 546 samples, 45 did not meet the inclusion criteria and were excluded from the study (Figure 2). Samples were categorised into five groups according to the SARS-CoV-2 immunisation status of the respective donors (naïve, vaccinated once, twice, or three times with mRNA-based vaccines, and COVID-19 convalescent patients). After the exclusion of 166 samples that did not fit the defined criteria, 335 samples from unique individuals were included in the analysis, of which 68 were naïve for SARS-CoV-2, 9 were vaccinated once, 141 were vaccinated twice, 55 were vaccinated three times, and 62 were COVID-19 convalescent (Figure 2).

Table 1 presents the characteristics of study participants included in the diagnostic performance analysis. Median (range) age was 49.5 (18–87) years for the naïve group, 43 (30–61) years, 40 (20–86) years, and 43 (21–67) years for the 1×-, 2×-, and 3×-vaccinated groups, respectively, and 38.5 (20–68) years for the COVID-19 convalescent group. 58.8% of naïve participants, 77.8%, 70.2%, and 69.1% of 1×-, 2×-, and 3×-vaccinated participants, and 66.1% of convalescent participants were female. The median (range) time since vaccination was 20 (16–35), 97 (17–197), and 32 (15–190) days for 1×-, 2×-, and 3×-vaccinated participants, respectively. The median (range) time since SARS-CoV-2 diagnosis in COVID-19 convalescent patients was 62 (17–240) days.

### 3.2. Diagnostic Performance of T-Track^®^ SARS-CoV-2

**Figure 3:**
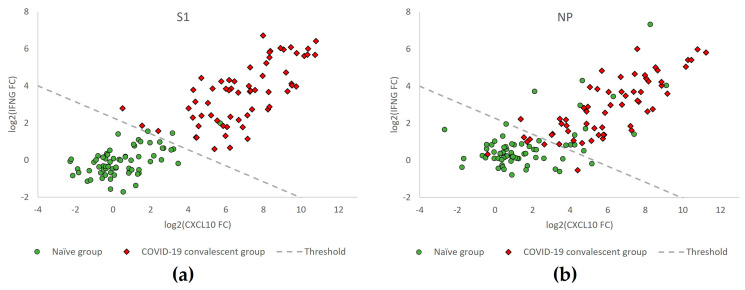
*IFNG* and *CXCL10* mRNA expression levels in samples from donors of the naïve versus the COVID-19 convalescent groups (*n* = 68 vs. *n* = 62) obtained with T-Track^®^ SARS-CoV-2 reactive for S1 (**a**) and for NP (**b**) antigens. Scatter plot showing log2-transformed fold change (FC) values of *IFNG* and *CXCL10* mRNA levels in samples from COVID-19 convalescent (red diamonds) and naïve participants (green circles). The dashed line (classification threshold) classifies the samples into “positive” (upper right) and “negative” (lower left).

68 donors naïve for SARS-CoV-2 (SARS-CoV-2-negative) and 62 COVID-19 convalescent participants (SARS-CoV-2-exposed) were investigated for T cell reactivity against the SARS-CoV-2 antigens S1 and NP. Changes in levels of *IFNG* and *CXCL10* mRNA (log2-transformed *IFNG* and *CXCL10* fold change (FC) values) in these samples are represented in Figure 3 to illustrate the discrimination of naïve (green circles) from COVID-19 convalescent (red diamonds) participants relative to the classification threshold (dashed line).

The diagnostic performance of T-Track^®^ SARS-CoV-2 in response to the respective S1 and NP SARS-CoV-2 antigens to discriminate naïve from COVID-19 convalescent donors is shown in Table 2. Moreover, 62/62 (100%) of the COVID-19 convalescent donors responded to the S1 protein, while 55/62 (88.7%) showed a response to NP. Among the 68 naïve donors, 3 (4.4%) responded to the S1 protein, while 13 (19.1%) showed NP reactivity. The invalid and inconclusive rates were 0% in response to both antigens (Table 2).

### 3.3. Age-Related Performance of T-Track^®^ SARS-CoV-2

**Figure 4:**
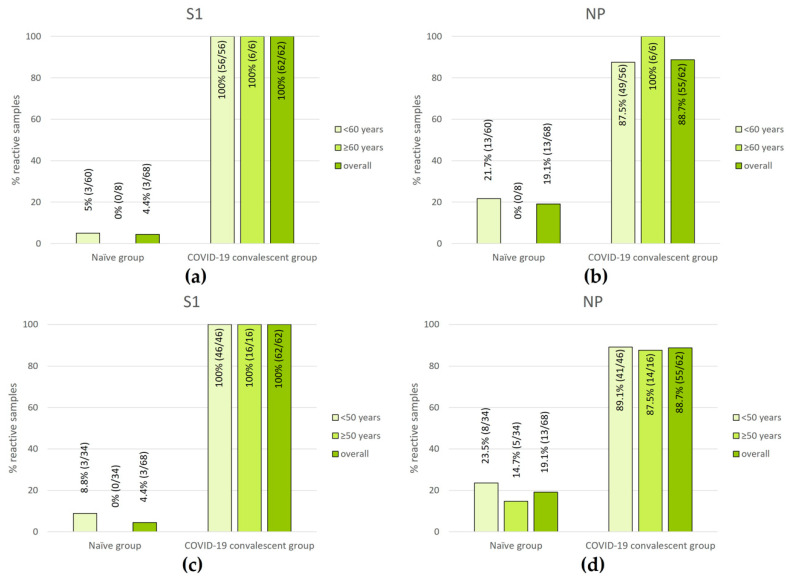
Percentages of donor samples with S1- or NP-reactive cells using T-Track^®^ SARS-CoV-2 in naïve and COVID-19 convalescent participants, overall (all age groups) and according to age distribution: <60 and ≥60 years (**a**,**b**) and <50 and ≥50 years (**c**,**d**). The respective proportions and percentages of reactive samples are shown within the diagram bars.

Next, we investigated the influence of age on the strength of immune response (so-called “high-risk group for COVID-19”; [20,35,36]) by the T-Track^®^ SARS-CoV-2 assay. To this aim, the response to S1 and NP antigens was measured in naïve vs. convalescent donors <60 and ≥60 years of age (Figure 4a,b) and <50 and ≥50 years of age (Figure 4c,d).

As to S1 reactivity, 100% of COVID-19 convalescent donors were responsive, regardless of their age category (Figure 4a,c). Similarly, the reactivity to the NP antigen in COVID-19 convalescent donors was high across all age groups, ranging from 87.5% to 100% (Figure 4b,d).

In older naïve donors, the apparent lower cross-reactivity was not significant. This trend was observed for donors ≥60 years of age (0/8 (0%) for both S1 and NP, compared to 3/60 (5.0%) in response to S1 and 13/60 (21.7%) in response to NP in donors <60 years) (Figure 4a,b), but also in donors ≥50 years of age (0/34 (0%) vs. 3/34 (8.8%) for donors ≥50 vs. <50 years in response to S1; 5/34 (14.7%) vs. 8/34 (23.5%) for donors ≥50 vs. <50 years in response to NP) (Figure 4c,d).

### 3.4. Persistence of SARS-CoV-2-Specific Humoral and Cellular Immune Responses

To investigate the persistence of T cell- and antibody-mediated immunity following SARS-CoV-2 immunisation (by vaccination or infection), T-Track^®^ SARS-CoV-2 and recomLine SARS-CoV-2 IgG test results were analysed in vaccinated and/or COVID-19 convalescent donors according to the time from the last recorded exposure to S1 or NP antigen. The last recorded antigen exposure was defined as the last vaccination episode (using spike mRNA-based vaccine) in the case of vaccinated donors (thus, reflecting the last exposure to S1 antigen) and as the infection diagnosis in COVID-19 convalescent donors (reflecting the last exposure to S1 and NP antigens). This analysis included participants with a blood withdrawal beyond the time frame of 14–275 days (after the last antigen exposure), and only samples with valid results for both T-Track^®^ SARS-CoV-2 and recomLine SARS-CoV-2 IgG tests were included.

**Figure 5:**
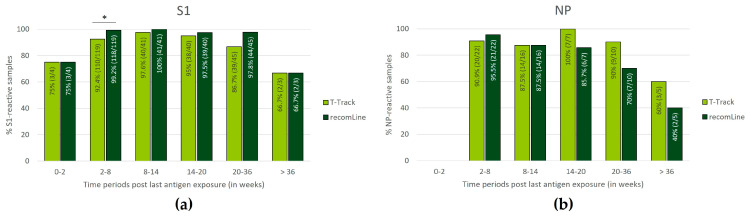
Distribution of samples reactive to S1 (**a**) or NP (**b**) antigen using T-Track^®^ SARS-CoV-2 and recomLine SARS-CoV-2 IgG assays according to the time from the last antigen exposure (defined time periods of 0–2, 2–8, 8–14, 14–20, 20–36, and >36 weeks post last antigen exposure). In (**a**), the analysis included donors immunised with spike mRNA-based vaccines (1×-, 2×-, 3×-vaccinated) or by SARS-CoV-2 infection (COVID-19 convalescent donors), thus exposed to S1 antigen. In (**b**), the analysis included COVID-19 convalescent donors only (exposed to NP antigen). Only samples with valid results for both T-Track^®^ SARS-CoV-2 and recomLine SARS-CoV-2 were included in the analysis. In (**a**,**b**), the proportions and percentages of reactive samples are indicated within the diagram bars. * p < 0.05.

The evaluation of S1-reactive samples was conducted in 1×-, 2×-, and 3×-vaccinated donors and in COVID-19 convalescent donors. If a convalescent donor was vaccinated after infection diagnosis, the date of vaccination was used as the last recorded S1 antigen exposure. In addition to the 267 vaccinated and convalescent donors included in the previous analysis (Figure 2 and Table 1), 10 further donors were included due to the unrestricted time window, and 9 samples with invalid T-Track^®^ SARS-CoV-2, 1 sample with inconclusive T-Track^®^ SARS-CoV-2 and 15 samples without recomLine SARS-CoV-2 IgG results were excluded. Thus, 252 samples were included in the analysis of S1-reactive samples according to the time from the last known S1 antigen exposure (Figure 5a).

The evaluation of NP-reactive samples was conducted in COVID-19 convalescent donors. In addition to the 62 convalescent donors included in the previous analysis (Figure 2 and Table 1), 6 further donors were included due to the unrestricted time window, and 1 sample with invalid T-Track^®^ SARS-CoV-2 result and 7 samples without recomLine SARS-CoV-2 IgG results were excluded. Thus, 60 samples were included in the analysis of NP-reactive samples according to the time from the last recorded NP antigen exposure (Figure 5b).

Within the first two weeks of immunisation, 3/4 (75.0%) of the donors showed S1-reactive T-Track^®^ SARS-CoV-2 and recomLine SARS-CoV-2 IgG results (Figure 5a). No NP reactivity data were available for this period (Figure 5b). Between 2 and 36 weeks post-last immunisation, the percentage of S1- and NP-reactive T-Track^®^ SARS-CoV-2 results were high, ranging from 86.7% to 100% (Figure 5a,b). Beyond 36 weeks, 2/3 (66.7%) and 3/5 (60.0%) samples were reactive to S1 and NP, respectively, using T-Track^®^ SARS-CoV-2 (Figure 5a,b). The percentage of S1-reactive recomLine SARS-CoV-2 IgG results was also high between 2 and 36 weeks after the last immunisation (ranging from 92.2% to 100%; Figure 5a), while the percentage of NP-reactive recomLine SARS-CoV-2 IgG results was elevated between 2 and 20 weeks (ranging from 85.7% to 95.5%; Figure 5b). Beyond 36 weeks (S1 reactivity) and 20 weeks (NP reactivity), the percentage of positive recomLine SARS-CoV-2 IgG results were lower (2/3 (66.7%) S1-reactive after 36 weeks, 7/10 (70.0%) NP-reactive at 20–36 weeks, and 2/5 (40.0%) NP-reactive after 36 weeks; Figure 5a,b).

### 3.5. Impact of Number of mRNA Vaccinations on T Cell Reactivity

**Figure 6:**
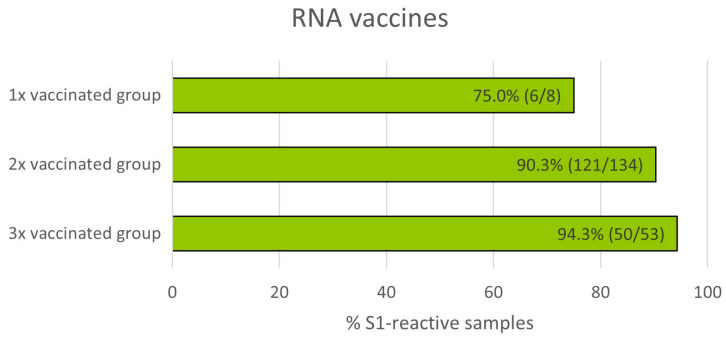
Percentage of S1-reactive cells measured by T-Track^®^ SARS-CoV-2 in donors immunised with one, two, or three doses of spike mRNA vaccine.

Next, T-cell-mediated immune responses measured by T-Track^®^ SARS-CoV-2 were compared in donors who had received one, two, or three doses of mRNA vaccine. Of the 205 samples from vaccinated donors (Figure 2 and Table 1), 9 with invalid and 1 with inconclusive T-Track^®^ SARS-CoV-2 results were excluded. Therefore, a total of 195 samples were included in this analysis, of which 8 donors were vaccinated once, 134 were vaccinated twice, and 53 were vaccinated three times (Figure 6). As expected, the proportion of S1-reactive donors increased with the number of vaccinations (from 75.0% to 94.3%).

### 3.6. ROC Curve Analysis Confirms Enhanced Performance by Dual-Marker Analysis

**Figure 7:**
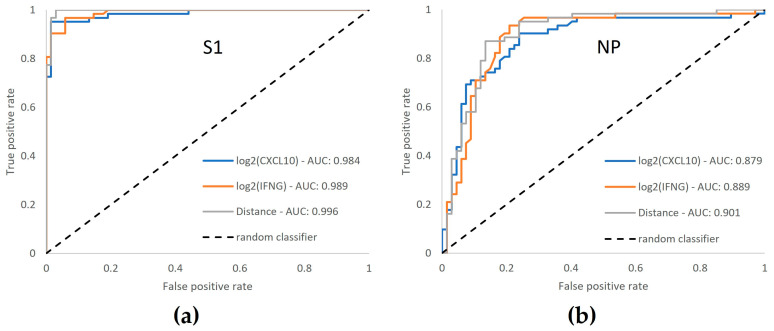
Receiver operating characteristic (ROC) curves for T-Track^®^ SARS-CoV-2 in response to S1 (**a**) and NP (**b**) antigens. The classification of 68 naïve and 62 convalescent donors was performed based on single log2-transformed *CXCL10* and log2-transformed *IFNG* FC values as well as on dual-marker evaluation (distance to the classification threshold). The true positive rate (*Y* axis) reflects the detection of reactive cells in COVID-19 convalescent donors, while the false positive rate (*X* axis) reflects the detection of reactive cells in donors naïve for SARS-CoV-2. AUC, area under the curve.

To confirm the benefit of the dual-marker evaluation of T-Track^®^ SARS-CoV-2 (based on both *IFNG* and *CXCL10*), we compared the performance of the single vs. combined markers to detect S1- and NP-reactive cells. Single log2-transformed fold change (FC) values as well as combined evaluation of both markers (distance of measured data to the classification threshold) of T-Track^®^ SARS-CoV-2 results from 62 COVID-19 convalescent and 68 naïve donors were used for ROC curve analyses (Figure 7).

For the determination of both S1- and NP-reactive samples, area under the curve (AUC) values were higher for dual-marker evaluations than for the respective single-marker evaluations (0.996 vs. 0.984 and 0.989 for S1 (Figure 7a); 0.901 vs. 0.879 and 0.889 for NP (Figure 7b)).

The highest Youden index for dual-marker analysis, which reflects the optimal diagnostic performance (i.e., allowing the best discrimination of convalescent and naïve donors), was 0.971 for S1 reactivity, with 100% S1-reactive COVID-19 convalescent donors and 2.9% S1-reactive naïve donors, and 0.737 for NP reactivity, with 87.1% NP-reactive COVID-19 convalescent donors and 13.4% NP-reactive naïve donors.

### 3.7. Separation of T-Track^®^ SARS-CoV-2 Test Results Relative to the Classification Threshold, According to Donors’ SARS-CoV-2 Exposure Status

**Figure 8:**
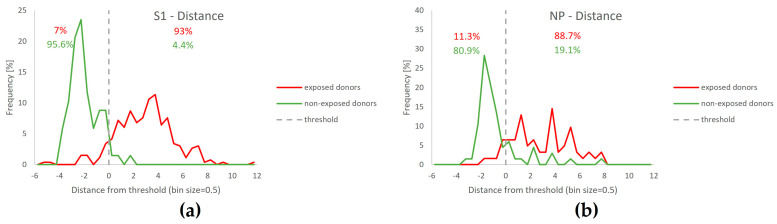
Distribution of T-Track^®^ SARS-CoV-2 distance values (distance to classification threshold) for S1 (**a**) and NP (**b**) reactivity in donors previously exposed to SARS-CoV-2 (red line) vs. those with no known exposure to SARS-CoV-2 (green line).

To illustrate the reliability of the T-Track^®^ SARS-CoV-2 interpretation algorithm, the distribution of the distance to the classification threshold was evaluated in samples of donors exposed to SARS-CoV-2 (through infection or vaccination) and not exposed to SARS-CoV-2 (naïve), in response to S1 or NP antigens (Figure 8). For S1-stimulated assays, 68 naïve donors (Figure 8a, green line), 195 spikes mRNA-vaccinated, and 62 COVID-19 convalescent donors (Figure 8a, red line) were evaluated. For NP-stimulated assays, 68 naïve donors (Figure 8b, green line) and 62 COVID-19 convalescent donors (Figure 8b, red line) were evaluated. In both antigen-stimulated samples, most SARS-CoV-2-exposed donors (93.0% for S1, 88.7% for NP; Figure 8a,b) discriminated well from the classification threshold. Naïve donors discriminated better from the classification threshold in S1-stimulated samples (95.6%; Figure 8a) than in NP-stimulated samples (80.9%; Figure 8b). Overall, the T-Track^®^ SARS-CoV-2 algorithm discriminated well between donors exposed to SARS-CoV-2 and those with no known exposure to SARS-CoV-2.

## 4. Discussion

This study evaluated several performance parameters of the novel commercially available T-Track^®^ SARS-CoV-2 test. T-Track^®^ SARS-CoV-2 is an RT-qPCR-based assay monitoring T cell responses to SARS-CoV-2 antigens. Compared to conventional IGRAs, T-Track^®^ SARS-CoV-2 combines the advantages of a more sensitive RT-qPCR method and the measurement of *CXCL10* expression in addition to *IFNG*. CXCL10 is produced in large quantities by antigen-presenting cells, particularly monocytes, in response to IFN-γ signaling, thereby enhancing the IFN-γ response of T cells to antigenic stimulation. Just recently, Schwarz et al. [37] demonstrated the suitability of the mRNA biomarker *CXCL10* in the development of a rapid, scalable assessment of SARS-CoV-2 cellular immunity by whole-blood quantitative PCR upon stimulation with selected spike peptides. IFN-γ is secreted by the vast majority of SARS-CoV-2-reactive Th-cells and CTL in SARS-CoV-2 infected and vaccinated individuals [16,38].

The benefit of combining *IFNG* and *CXCL10* over single markers was indeed confirmed in our ROC analysis. The implementation of a threshold-based algorithm further allows the reliable discrimination of reactive and non-reactive samples, as previously demonstrated for the T-Track^®^ TB assay [32]. In addition, the T-Track^®^ SARS-CoV-2 assay provides the possibility to detect T cells reactive to the SARS-CoV-2 S1 subunit of the spike protein (S1) and the nucleocapsid protein (NP) separately, which may be relevant for studies investigating cross-reactivity, clinical protection, and responses to vaccines [4,18]. In addition to the M protein (membrane protein), S and N antigens have been described as the major targets of SARS-CoV-2-reactive T cells [15,19]. Indeed, based on the currently authorized SARS-CoV-2-specific vaccines, which rely on the spike protein as an immunogen, T-Track^®^ SARS-CoV-2 assay is expected to detect T cells reactive to the S1 antigen in all vaccinated individuals, in addition to subjects with a history of SARS-CoV-2 infection. By contrast, non-vaccinated individuals with past exposure to SARS-CoV-2 are expected to present T cells reactive to the NP antigen and thus produce an NP-specific response with T-Track^®^ SARS-CoV-2. Our data suggest that NP immunogenicity is weaker than S1 immunogenicity in terms of cellular immunity in COVID-19 convalescents, with a reactivity of 88.7% to NP versus 100% to S1 in the same individuals. Moreover, the NP full-length protein used in the T-Track^®^ SARS-CoV-2 assay is predicted to reveal T cell responses to past exposure to the seasonal human coronaviruses (HCoV), including in subjects naïve for SARS-CoV-2. This cross-reactivity associated with the SARS-CoV-2 NP antigen likely explains the higher reactivity rate to NP in naïve participants (19.1% reactivity to NP vs. 4.4% reactivity to S1). This agrees with reports indicating that 35–60% of individuals never exposed to SARS-CoV-2 (via infection or vaccination) have T cells cross-reactive to SARS-CoV-2 [15,18,19]. Such cross-reactivity is clinically relevant, as there is increasing evidence that it may be important in clinical protection against SARS-CoV-2. Recent exposure to seasonal HCoV and the following generation of memory T cell responses, with a cross-protective potential against SARS-CoV-2, appear to be associated with a better clinical outcome after SARS-CoV-2 infection [1,2,3,4]. Thus, by allowing an individual evaluation of the response to S1 and NP, T-Track^®^ SARS-CoV-2 could be suitable for cross-reactivity and protection studies. On the other hand, although only donors with no known history of SARS-CoV-2 exposure and negative anti-S1 and anti-NP recomLine SARS-CoV-2 IgG results were included in this study, a previous undetected SARS-CoV-2 infection cannot be totally excluded in our naïve population, as antibodies wane or may become undetectable over time [38,39].

The age-associated decline of immunity is a serious health concern. In general, impaired T-cell response in older people is associated with reduced immunogenicity and reactogenicity of mRNA COVID-19 vaccination [40]. People aged 60 years and older are generally included in the “high-risk group for COVID-19” [41,42]. Currently, the most comprehensive report on risk factors for death due to COVID-19 includes data from more than 44,000 confirmed individuals from the Chinese Center for Disease Control and Prevention [43]. This observational study showed a case fatality rate of 0.2–0.4% in infected people <50 years vs. 1.3% in infected people 50–59 years of age. For donors ≥60 years, a very good performance was observed for T-Track^®^ SARS-CoV-2, with 100% of the convalescent donors responding to S1 and NP and 0% of the naïve donors being responsive to both antigens. However, these results should be taken with caution, given the small sample size of naïve (*n* = 8) and convalescent (*n* = 6) participants ≥60 years. For donors ≥50 years, a comparable reactivity to S1 was observed (in 100% convalescent and 0% naïve donors). In comparison, reactivity to NP was lower than to S1 in convalescent donors ≥50 years (87.5% vs. 100%), while it was higher in naïve donors ≥50 years (14.7% vs. 0%). On the other hand, since negative S1 and NP results for recomLine SARS-CoV-2 IgG were inclusion criteria for the naïve group, and antibody titers are known to decrease over time [9,10,11], while SARS-CoV-2-specific T cells can be observed in the absence of SARS-CoV-2-specific antibodies [12,13,14], an undetected past infection (without a history of PCR positivity) can never be totally excluded [38,39]. Furthermore, we observed a trend to lower S1 and NP reactivity in older naïve donors (0% vs. 8.8% for S1 and 14.7% vs. 23.5% for NP in donors ≥50 vs. <50 years). Besides a reduced immune reactivity associated with age, we cannot exclude the possibility of a changed contact behaviour of older people (e.g., self-chosen isolation) and, therefore, fewer infection cases among older people [44]. In accordance with this assumption, younger people are at higher risk of obtaining a SARS-CoV-2 infection due to their higher contact rate, notably with children. Nevertheless, T-Track^®^ SARS-CoV-2 showed comparable performance data in younger and older COVID-19 convalescent donors (<50 and ≥50 years) and could, therefore, be used without restrictions in persons of all ages. Future studies should evaluate the performance of T-Track^®^ SARS-CoV-2 in other high-risk groups for COVID-19, such as immunocompromised patients.

Because of reported differences in the persistence of antibody-mediated and T cell-mediated immunity over time [9,10,11,12,13,14], we compared T-Track^®^ SARS-CoV-2 results to those of recomLine SARS-CoV-2 IgG in the same individuals at various time frames after the last known SARS-CoV-2 antigen exposure. The overall patterns of test positivity per time period for T-Track^®^ SARS-CoV-2 and recomLine SARS-CoV-2 IgG were similar. Reactivity to S1 increased after 2 weeks of the last antigen exposure and decreased after 36 weeks, while reactivity to NP decreased after 20 weeks of antigen exposure. Antibody response to NP seemed to be weaker than the respective T cell response 36 weeks after the last immunisation. However, results in this time window should be taken with caution due to the small sample size (*n* = 5) and the resultant missing significance. If confirmed in a larger cohort, this result would confirm the observation that SARS-CoV-2-specific antibodies wane over a period of months after infection [9,10]. Noteworthy, questions remain as to whether the presence of SARS-CoV-2 antibodies protects against SARS-CoV-2 infection or if it can be used for prognosis of the clinical course of COVID-19 since COVID-19 convalescent persons with undetectable antibodies showed T cell immunity against SARS-CoV-2 [12,13,14]. Typically, IgA, M, and G antibodies play a dominant role in SARS-CoV-2-responsive humoral immune responses, with inflammatory IgG1 being the most abundant [45]. Notably, recent studies from Irrgang and co-workers demonstrated a class switch towards non-inflammatory, spike-specific IgG4 antibodies after repeated SARS-CoV-2 mRNA vaccination, strongly suggesting an altered immunoprotective capacity of humoral immunity [46].

In contrast to short-lived antibodies, SARS-CoV-2-specific T-cell immunity has been reported to be maintained for at least 12 months after infection [30,31]. The long-term durability of T-cell responses against SARS-CoV-2 is unknown, but it was shown that T-cell responses against SARS-CoV-1 can be elicited even 6 to 17 years after infection [11,19]. Because the results of samples tested side-by-side with recomLine SARS-CoV-2 IgG and T-Track^®^ SARS-CoV-2 showed a good general concordance, T-Track^®^ SARS-CoV-2 could represent an additional tool alongside antibody testing for monitoring SARS-CoV-2-specific immune responses. Interestingly, the analysis of T cell responses by the T-SPOT^®^.COVID assay (Oxford Immunotec) in patients with a confirmed SARS-CoV-2 infection using similar time windows as in our study revealed similar patterns of T-cell response [25].

T cell-mediated immune responses measured by T-Track^®^ SARS-CoV-2 in donors who had received multiple doses of mRNA vaccine were boosted, confirming existing evidence of the importance of T cell responses in vaccine-induced protection [3,7,47]. However, the value of this analysis is limited by the small sample size of the 1×-vaccinated group (*n* = 8) and the fact that blood withdrawal after vaccination was made at different time points (i.e., not synchronised; Table 1). The impact of vaccination on the robustness and durability of T-cell responses could be addressed by T-Track^®^ SARS-CoV-2 in future COVID-19 vaccination trials.

This study presents several limitations, including the definition of naïve donors based on negative serology, due to possible antibody waning several months after infection, the heterogeneous SARS-CoV-2-exposed donor groups for S1- and NP-specific responses according to the time post antigen exposure (vaccinated and convalescent vs. convalescent only, respectively), the small sample size in some of the presented analyses, and the non-standardised time of blood withdrawal following vaccination.

## 5. Conclusions

The T-Track^®^ SARS-CoV-2 assay showed an excellent performance in naïve and COVID-19 convalescent donors, including in donors ≥50 years of age. The performance evaluation according to time post immunisation revealed sustained positivity rates over several months after the last recorded S1 or NP antigen exposure. The main advantage of T-Track^®^ SARS-CoV-2 remains the dual-marker system for improved performance and the possibility to determine the NP reactivity, which could provide hints for cross-reactive T cells. Thus, T-Track^®^ SARS-CoV-2 is a sensitive and specific tool for monitoring T-cell responses elicited by SARS-CoV-2 infection or COVID-19 vaccination.

## Figures and Tables

**Table 1:** Characteristics of study participants.

Characteristics	Study Group				
	Naїve	1× Vaccinated	2× Vaccinated	3× Vaccinated	COVID-19 Convalescent
Study population, *N* (%)	68 (100%)	9 (100%)	141 (100%)	55 (100%)	62 (100%)
Age in years, median (range)	49.5 (18–87)	43 (30–61)	40 (20–86)	43 (21–67)	38.5 (20–68)
Sex, *N* (%)					
Female	40 (58.8%)	7 (77.8%)	99 (70.2%)	38 (69.1%)	41 (66.1%)
Male	28 (41.2%)	2 (22.2%)	42 (29.8%)	17 (30.9%)	21 (33.9%)
Time since diagnosis in days, median (range)	n.a.	n.a.	n.a.	n.a.	62 (17–240)
Time since last immunisation in days, median (range)	n.a.	20 (16–35)	97 (17–197)	32 (15–190)	n.a.

Abbreviation: n.a., not applicable.

**Table 2:** Percentage of donor samples with S1- or NP-reactive cells in 68 naïve and 62 COVID-19 convalescent donors measured by T-Track^®^ SARS-CoV-2. This table reflects the FC values depicted in Figure 3.

Antigen	Samples with Reactive T Cells, *n*/*N* (%) (95% CI)		Invalid Rate *n*/*N* (%)	Inconclusive Rate *n*/*N* (%)
	Naїve Group	COVID-19 Convalescent Group		
S1	3/68 (4.4%) (1.0–12.7)	62/62 (100.0%) (93.0–100.0)	0/130 (0.0%)	0/130 (0.0%)
NP	13/68 (19.1%) (11.4–30.1)	55/62 (88.7%) (78.2–94.7)	0/130 (0.0%)	0/130 (0.0%)

## Data Availability

The data presented in this study are available within the article.
